# Exploration of Appetite Regulation in Yangtze Sturgeon (*Acipenser dabryanus*) During Weaning

**DOI:** 10.3390/ijms26030950

**Published:** 2025-01-23

**Authors:** Bin Wang, Ni Tang, Shuhuang Chen, Xin Zhang, Defang Chen, Zhiqiong Li, Bo Zhou

**Affiliations:** 1 Fisheries Institute, Sichuan Academy of Agricultural Sciences, Chengdu 611731, China; wangbin@scsaas.cn (B.W.); tangni92sau@scsaas.cn (N.T.); 2 Fish Resources and Environment in the Upper Reaches of the Yangtze River Obervation and Research Station of Sichuan Province, Yibin 644000, China; 3 Department of Aquaculture, College of Animal Science and Technology, Sichuan Agricultural University, Chengdu 611130, China; 1124062356@sicau.edu.cn (S.C.); 14769@sicau.edu.cn (X.Z.); 13908@sicau.edu.cn (D.C.)

**Keywords:** *Acipenser dabryanus*, transcriptome, appetite regulation, weaning

## Abstract

Yangtze sturgeon is an endangered fish species. After weaning, some Yangtze sturgeon fry refuse to consume any food, which causes a low survival rate during the artificial breeding period. This study showed that the body length and body weight of failed weaning Yangtze sturgeons were significantly lower than those of successful weaning sturgeons. Since the brain is the center of appetite regulation, RNA-seq of the brain was employed to analyze the differentially expressed genes and their biological functions in successfully and unsuccessfully weaned fry. After that, 82,151 unigenes and 3222 DEGs were obtained. Based on the results of RNA-seq, appetite factors, including *POMC*, *CART*, *NPY* and *AgRP*, were cloned, and then a weaning experiment was designed to explore the changes in appetite after feeding a microcapsule diet (weaning group). The results showed that, during the weaning period, the expression of *CART* was increased on the 1st and 3rd days but decreased on the 5th, 6th, 8th and 10th days. The expression of *AgRP* was downregulated on the 1st and 3rd days but upregulated on the 5th, 6th, 8th and 10th days. These findings indicate that appetite was suppressed in the early and middle periods but enhanced in the latter period of weaning and that *CART* may play an important role in the appetite-suppressing effect.

## 1. Introduction

Aquaculture is the fastest growing food industry in the world [1]. With the development of aquaculture and the advancement of intensive farming, the demand for high-quality fry in aquaculture is increasing. The traditional food of the fry is live food, including rotifers (*Brachionus* spp.) and brine shrimp nauplii (*Artemia* spp.), which are used due to their high digestibility and eminent palatability [2,3]. However, previous studies have indicated that the nutritional value of live food is difficult to balance, especially the contents of limiting amino acids and unsaturated fatty acids, and the production procedures of live food are complicated [4,5]. In addition, live food is also a carrier of *Vibrio* spp., which will result in a high mortality of the larvae [6]. The replacement of live food with an artificial diet will meet the nutritional needs of the fry, reduce the cost in aquaculture and improve the survival rate during the larvae-rearing period. Therefore, replacing live food via a weaning procedure with an artificial diet is an important procedure. Nevertheless, the food intake of larvae will be decreased, and several larvae even take in nothing because of a lack of adaptation to the new diet [7]. To date, studies have mainly focused on the effects of weaning, such as the immunity, growth performance and digestive function of larvae [8,9,10,11], but information on the regulation of food intake during the period of weaning remains elusive.

Food intake is a complex process of balancing energy intake and consumption in the physiological system, which is also the consequence of a balance among hunger, appetite and satiety [12,13] and is primarily controlled by the central nervous system in animals. The proopiomelanocortin/cocaine and amphetamine transcription regulated transcript (POMC/CART) neurons and the neuropeptide Y/agouti-related protein (*NPY*/*AgRP*) neurons are the two critical appetite neuron populations in the arcuate nucleus [14]. Previous studies have shown that these neurons regulate the appetite of animals by releasing anorexigenic or orexigenic factors [15,16]. An increasing number of hormones involved in appetite regulation have been identified from fish species. Recent studies in mammals such as rats and piglets have found that several appetite factors in the brain were affected during weaning. Weaning SD rats fed a high-fat diet downregulated the expression of anorexia genes (*POMC*, *CART*) and upregulated the expression of anorexigenic genes (*NPY*, *AgRP*) [17]. The expression of NPY in the hypothalamus of weaning piglets was decreased significantly, and the expression of POMC and AgRP showed no significant change. In teleosts, only Chinese perch (*Siniperca chuatsi*) was reported to have downregulated expression of *POMC* and *leptin* in response to the replacement of live feed [18]. These studies indicate that the expression of appetite factors undergoes changes during weaning, potentially influencing the food intake of animals and thereby affecting the success of the weaning process, while there are still few relevant studies in aquatic animals. In addition, unlike mammals, the role of appetitive hormones in fish exhibits species specificity that may be associated with different feeding habits and evolutionary positions [19,20]. However, there are limited reports on the mechanism related to fish weaning. Therefore, it is necessary to investigate the detailed analysis of the appetite regulatory mechanism in fish during weaning.

The Yangtze sturgeon (*Acipenser dabryanus*) has been included on the Red List of the International Union for Conservation of Nature (IUCN) [21]. Due to overfishing, habitat deterioration and dam construction, the number of Yangtze sturgeon in the wild has decreased sharply in the past three decades. Since 1990, the Yangtze sturgeon has completely lost its natural reproduction [22,23], and artificial breeding is the only way to restore its natural population. The major obstacle to artificial sturgeon breeding is the reluctance of fry to accept an artificial microencapsulated diet [4]. Our group has long studied the feeding of fish and has reported several factors in sturgeon that promote or suppress food intake [24,25,26,27,28,29]. However, the changes in appetite and the mechanism of appetite regulation in weaning sturgeons are still unclear.

In the present study, RNA-seq of the Yangtze sturgeon brain was employed to explore the genes affected by weaning. Based on the results of RNA-seq, four appetite factors, *POMC*, *CART*, *NPY* and *AgRP*, were cloned, and their expression in the Yangtze sturgeon brain during different weaning periods was detected. The current study was conducted to explore the response of appetite regulatory factors and analyze the expression of these factors in different processes of weaning.

## 2. Results

### 2.1. Growth Performance

**Figure 1:**
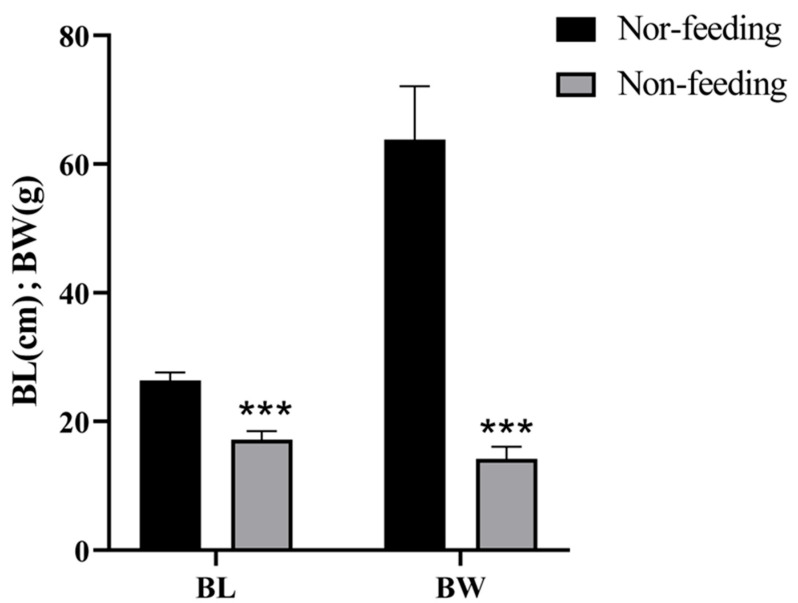
The body length and body weight of the sturgeons of successful (Nor-feeding) or failed (Non-feeding) weaning. The results are presented as mean ± SEM, the asterisk indicates a significant difference (*p* < 0.05).

The results showed that, 160 DPH, the body length (17.16 ± 1.25 cm) and body weight (14.19 ± 1.78 g) of non-feeding Yangtze sturgeons were significantly lower than those of normal feeding (nor-feeding) Yangtze sturgeons (26.36 ± 1.21 cm, 63.82 ± 7.87 g; *p* < 0.001, *p* < 0.001; Figure 1).

### 2.2. Illumina Sequencing and De Novo Transcriptome Assembly

This study determined the transcriptome of brain tissues of failed weaning and successful weaning Yangtze sturgeons at 160 DPH. The results showed that 62,910,056 and 53,290,740 raw reads were obtained in the F_B and S_B groups, respectively. Then, after quality filtering, 62,356,550 and 52,743,864 clean reads were obtained in the F_B and S_B groups, respectively, with Q20 (nucleotides with quality values > 20) > 98%, Q30 > 94% and error rate < 0.025% (Table S1). These clean reads were assembled into 126,855 transcripts with an N50 length of 1987 bp, and a final set of 82,151 unigenes was generated (Table S2). The scores for the quality assessment of transcriptome assembly using TransRate and BUSCO were 0.41746 and 89.7%**,** respectively.

### 2.3. Unigene Annotation

**Figure 2:**
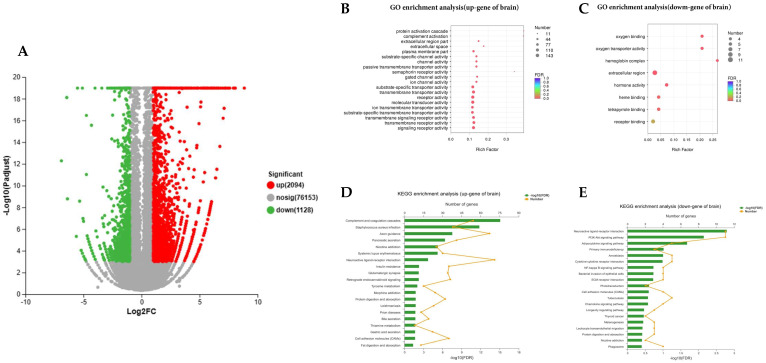
Upregulated or downregulated genes in brains of sturgeons of successful or failed weaning. (**A**) Volcano plots of differentially expressed unigenes in sturgeons of successful or failed weaning. (**B**,**C**) GO enrichment of upregulated or downregulated genes in brains, (**D**,**E**) KEGG pathway enrichment of upregulated or downregulated genes in brains.

To obtain annotation information, the 79,375 unigenes were annotated in six databases, including NR, SwissProt, Pfam, COG, GO and KEGG, after assembly optimization (Figure 2A). The results showed that 30,836 (38.85%), 23,833 (30.03%), 21,390 (26.95%), 26,041 (32.81%), 24,166 (30.45%) and 17,489 (22.03%) unigenes were annotated in the above databases, respectively (Figure S1A). In addition, 15,922 clusters (50.77%) were similar to those of *Acipenser ruthenus*, 4916 to those of *Lepisosteus osseus* (15.44%) and 3844 to those of *Erpetoichthys calabaricus* (12.26%) (Figure S1B).

### 2.4. Filtration and Annotation of Differentially Expressed Genes

In this study, |log2 (fold change)| ≥ 1 and *p*-adjust < 0.001 were taken as the threshold of differential expression levels. A total of 3222 differentially expressed unigenes (DEGs) were screened in the brain transcriptome. Specifically, S_B vs. F_B upregulated 2094 DEGs and downregulated 1128 DEGs (Figure 2A). Subsequently, GO and KEGG pathway enrichment analyses of these DEGs were performed.

**Figure 3:**
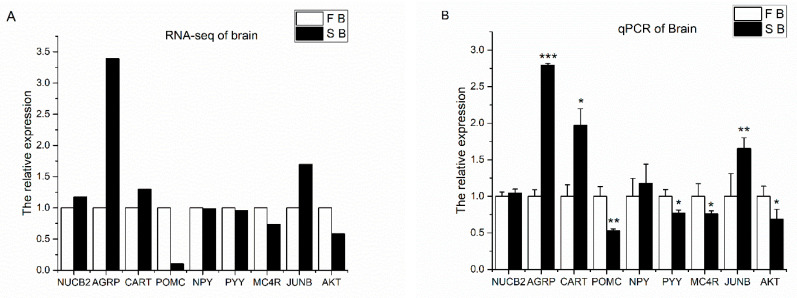
Validation of appetite-related genes of the transcriptome DEGs (**A**) in Yangtze sturgeon using qPCR (**B**). Gene expression levels were normalized to that of *β-actin* and *EF1-α*. Data are presented as the group means ± SEM (*n* = 5). Statistical comparison of the mRNA levels detected in different groups was carried out by *t*-test of variance (* *p* < 0.05, ** *p* < 0.01, *** *p* < 0.001).

The results of GO enrichment showed that a total of 1228 upregulated DEGs were enriched in 396 GO terms, of which 1135 DEGs were significantly enriched in 74 GO terms (*p* < 0.05, Figure 3B). The 15 biological process terms included protein activation cascade, complement activation and humoral immune response, extracellular region part and extracellular space, 10 cellular component terms included extracellular space and plasma membrane part, and 49 molecular function terms included substrate-specific channel activity, channel activity and receptor activity (Figure 2B). Moreover, 20 downregulated DEGs were enriched in 170 GO terms, of which 17 DEGs were significantly enriched in 7 GO terms (*p* < 0.05), including hemoglobin complex, oxygen binding and oxygen transporter activity (Figure 2C).

The results of KEGG annotation showed that a total of 1074 upregulated DEGs were enriched in 305 pathways, of which 435 DEGs were significantly enriched in 23 KEGG pathways (*p* < 0.05, Figure 2D), including complement and coordination cascades (map04610), axon guidance (map04360) and pancreatic secretion (map04972). In addition, 59 downregulated DEGs were enriched in 145 KEGG pathways, among which 24 DEGs were significantly annotated (*p* < 0.05, Figure 2E), including neuroactive ligand receptor interaction (map04080), PI3K/Akt signaling pathway (map04151) and adipocytokine signaling pathway (map04920).

### 2.5. Transcript Validation by qPCR

Several appetite factors and signal factors (*NUCB2*, *AgRP*, *CART*, *POMC*, *NPY*, *PYY*, *MC4R*, *JUNB*, *AKT*) were selected for qPCR based on the results of DEGs to validate the transcriptome data. The results showed that the expression patterns of these genes were similar to the detection of RNA-seq (Figure 3A,B).

### 2.6. Cloning and Sequence Analysis of Appetite Factors of Yangtze Sturgeon

To investigate the effects of weaning on appetite regulation in Yangtze sturgeon, *POMC*, *CART*, *NPY* and *AgRP* were cloned in this study according to the transcriptome results. The nucleotide sequences of *POMC*, *CART*, *NPY* and *AgRP* of Yangtze sturgeon were uploaded to GenBank with GenBank numbers MN685788, MN685803, MN685790 and MN685803.

**Figure 4:**
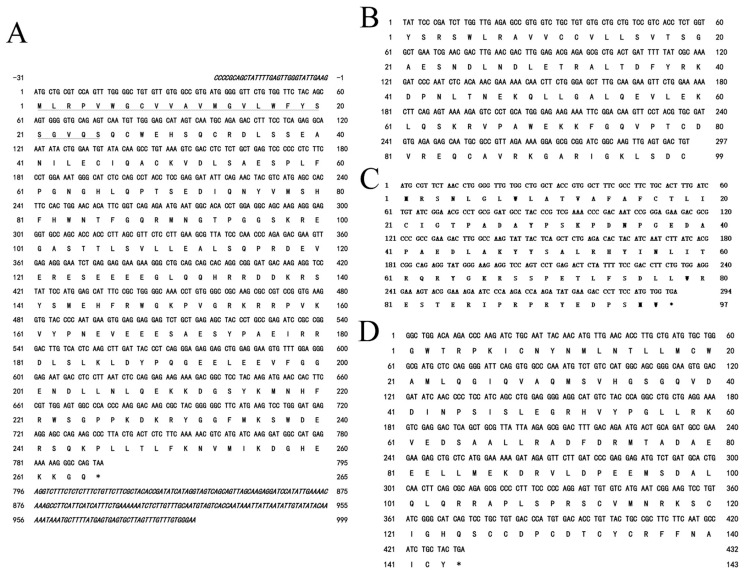
cDNA sequences and deduced amino acid sequences of *POMC* (**A**), *CART* (**B**), *NPY* (**C**), *AgRP* (**D**) in the Yangtze sturgeons. The putative signal peptide is underlined and the asterisk indicates the stop codon.

**Figure 5:**
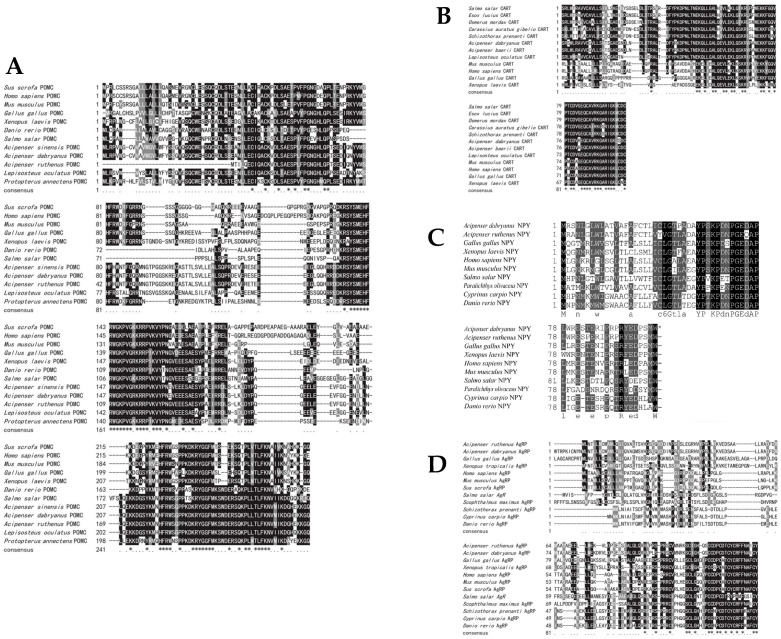
Alignment of the amino acid sequences of *POMC* (**A**), *CART* (**B**), *NPY* (**C**), *AgRP* (**D**). Different gray intensities indicate the conservation of the amino acids between species of the four genes. Identical residues are shaded in black, and residues shared by >33% of the sequences are shaded in gray.

The Yangtze sturgeon *POMC*, *CART*, *NPY* and *AgRP* nucleotide sequences were 1030 bp, 297 bp, 432 bp and 294 bp and encoded 261, 99, 97 and 141 amino acids, respectively (Figure 4). The present study was the first to obtain the cDNA sequence of Yangtze sturgeon *AgRP*, which encodes 141 amino acids. According to the multiple sequence alignment, the sequence of *POMC* in Yangtze sturgeon was the same as that in Chinese sturgeon, shared 84.3% similarity to *Acipenser ruthenus* and had the lowest similarity to humans (84.3%) (Figure 5A). The *CART* gene had the highest similarity to the Siberian sturgeon (98%), followed by *Lepisosteus oculatus* (82.65%) and the African clawed frog (*Xenopus laevis*) (68.18%) (Figure 5B). The *NPY* of Yangtze sturgeon and the *NPY* of *Acipenser ruthenus* are the most similar (98.97%), while showing the least similarity to that of zebrafish (64.95%) (Figure 5C). Finally, the consistency of the *CART* amino acid sequence between Yangtze sturgeon and Siberian sturgeon was the highest (98%), followed by spotted eel (83.2%), and the lowest was with African Xenopus, only 43.6% (Figure 5D).

**Figure 6:**
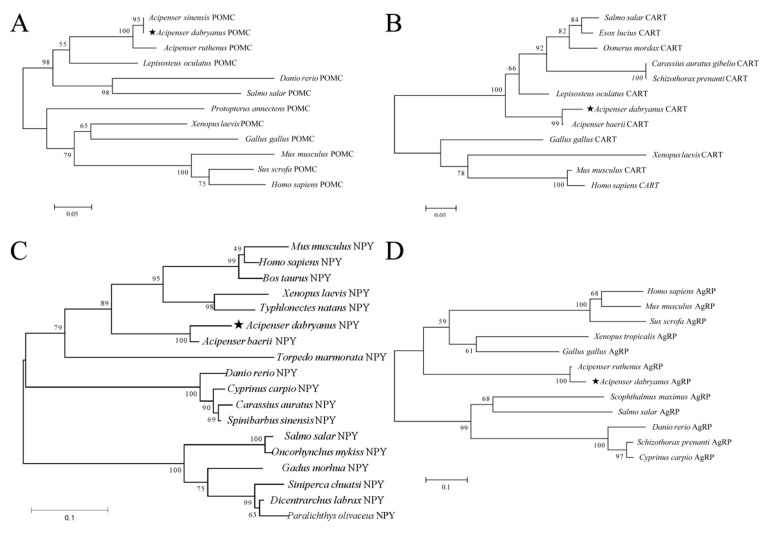
Phylogenetic trees of *POMC* (**A**), *CART* (**B**), *NPY* (**C**), *AgRP* (**D**) amino acid sequences in Yangtze sturgeon. Numbers at nodes indicate the bootstrap value, as percentages, obtained for 1000 replicates.

The results of the phylogenetic tree indicated that Yangtze sturgeon *POMC* first clustered Chinese sturgeon, then *Acipenser ruthenus*, and formed a large branch with spotted eel (*Gymnothorax melanospilus*), Ontario salmon (*Salmo salar*) and zebrafish (*Danio rerio*), while another large branch was formed with African lung fish (*Protopterus annectens*), mammals, birds, amphibians and bony fish (Figure 6A). The *CART* of Yangtze sturgeon was first clustered into one branch with Siberian sturgeon (*Acipenser baerii*), then into a large branch with other fish and finally its complete evolutionary tree was constructed with jungle fowl (*Gallus gallus*), African clawed frog (*Xenopus laevis*), mice (*Mus musculus*) and humans (*Homo sapiens*), which was consistent with animal classification (Figure 6B). The *NPY* of Yangtze sturgeon first converged with that of Siberian sturgeon (*Acipenser sinensis*), then converged with the branch formed by mammals, amphibians and reptiles and finally converged with that of other teleosts (Figure 6C). Finally, *AgRP* was divided into two branches, a fish branch and a non-fish branch. *AgRP* of Yangtze sturgeon and *Acipenser ruthenus* were clustered into one branch and then clustered in the non-fish branch (Figure 6D).

### 2.7. Effects of Weaning on the mRNA Expression of Appetite Factors in Yangtze Sturgeon

**Figure 7:**
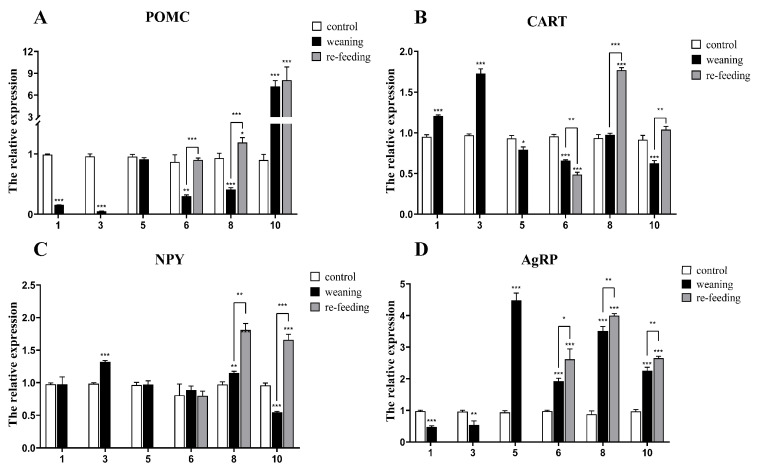
Weaning- and refeeding-induced changes in *POMC* (**A**), *CART* (**B**), *NPY* (**C**) and *AgRP* (**D**) mRNA abundances in the whole brain. Gene expression levels were normalized to that of *β-actin* and *EF1-α*. Data are presented as the group means ± SEM (*n* = 5). Statistical comparison of the mRNA levels detected in different groups was carried out by *t*-test of variance (* *p* < 0.05, ** *p* < 0.01, *** *p* < 0.001).

qPCR was conducted to investigate the changes in the mRNA expression of appetite factors during weaning. Compared with the control group, the appetite suppressor *POMC* in the weaning group decreased significantly on days 1, 3, 6 and 8, and there was no significant difference on day 5. After the 5th day, compared with the expression of the weaning group, the expression of *POMC* increased. At 10 days of weaning, it was elevated more than 5-fold (Figure 7A). The results showed that *CART* mRNA expression was elevated in the brains of weaning fish compared with fish in the control group on the 1st and 3rd days (Figure 7B). However, the *CART* levels in weaning fish showed a statistically significant decrease compared with control fish on the 5th, 6th and 10th days (Figure 7B). When fish were refed with Tubificidae, *CART* mRNA expression significantly increased compared with that in weaning fish on the 8th and 10th days (Figure 7B) but decreased on the 6th day. During the process of weaning, the expression of *NPY* changed slightly (Figure 7C) compared with the control group. *NPY* in the weaning group increased significantly only on the 3rd and 8th days (*p* < 0.01) and decreased significantly on the 10th day (*p* < 0.001), and there was no significant difference on other days of weaning, but it increased significantly on the 8th and 10th days after refeeding (Figure 7C, *p* < 0.01). In the weaning group, the level of *AgRP* decreased significantly in the early stage (1st and 3rd days) and increased in the middle and late stages (5th to 10th days). After refeeding, the level of *AgRP* was significantly (*p* < 0.05) elevated compared with the control and weaning groups (Figure 7D).

## 3. Discussion

Weaning is a critical stage in fish farming. In Yangtze sturgeon larval production, the growth rate in the Yangtze sturgeon that failed to wean is low, and some sturgeon refuse to consume the microencapsulated diet. To understand the mechanism of dietary change in the growth and metabolism of larval sturgeon, RNA-seq was performed to determine the transcription profile in the brain of Yangtze sturgeon. Several studies in teleosts have investigated the mRNA expression of the liver in weaning teleosts. Peng et al. [18] found that 24,819 genes were differentially expressed between mandarin fish fed normal feed and dead prey fish. Kobayashi et al. [30] obtained 42,631 unigenes and 867 DEGs in the liver of bass at different stages of weaning. The present results indicated that there were 126,855 transcripts and 82,151 unigenes, of which 3222 were DEGs. Our current study is the first to report the RNA-seq of the brain in weaning teleosts. The expression of the four candidate genes was determined by qPCR.

The KEGG enrichment showed that complement and coagulation cascade, Staphylococcus aureus and axon guidance were the main upregulated KEGG pathways in the failed weaning sturgeons, and neuroactive ligand-receptor interaction, PI3K-Akt signaling pathway and adipocytokine signaling pathway were the main downregulated KEGG pathways. Studies in mammals have also found that changes in food after weaning affect the expression of genes in the complement and coagulation cascade and Staphylococcus aureus pathways, including complement C3, C4 and IL-10. Duan et al. found that the expression of C3, C4 and IGA in piglets was upregulated after weaning [31], and Ma et al. found that a lack of magnesium or supplementation of zinc after weaning led to increased C3 levels in the plasma of mice and piglets [32,33], which was consistent with our results. An increasing number of studies have found that the PI3K pathway and adipocyte cytokine pathway can regulate appetite. PI3K can participate in POMC-, NPY- and CART-related appetite regulation [34,35]. Our previous studies have also confirmed that several adipocytokines play important roles in the regulation of sturgeon appetite [36]. Thus, the current results suggest that weaning may affect the appetite of sturgeon. Moreover, KEGG enrichment analysis showed that weaning also affects axon guidance pathways. Studies have shown that the appetite factors AgRP and POMC work through the projection of axons and regulate long-term appetite and short-term appetite [37]. Weaning also inhibits the neuroreceptor and ligand interaction pathway, including neuropeptide Y. In our previous research, we also found that it is a key factor in appetite regulation. CART expression was also upregulated in this study (*p* value < 0.005). However, since CART is not listed in any KEGG pathways, there are no CART-related pathways in the KEGG enrichment. In conclusion, due to the observation that the food intake of failed weaning Yangtze sturgeon may be reduced and that the transcriptomic results also suggested that the appetite of Yangtze River sturgeon changed, we conducted further studies on *POMC*, *CART*, *NPY* and *AgRP* in subsequent experiments.

POMC is the neuropeptide secreted by POMC/CART neurons. In the fasting experiment, the expression level of *POMC* mRNA in the brains of medaka, Atlantic salmon and channel catfish decreased. After feeding, the expression level of *POMC* mRNA in the brain of goldfish increased, suggesting that *POMC* is involved in the regulation of fish feeding [38]. Insulin promotes its translocation into the nucleus, binds to the *POMC* promoter, upregulates *POMC* expression and reduces food intake [39]. In the study of fish, the *CART* gene is highly expressed in the central nervous system, especially in the region related to appetite regulation in the hypothalamus [40,41,42,43]. The expression of *CART* in the hypothalamus of goldfish increased significantly after feeding for 2 h, while its expression decreased significantly by fasting [40]. Similar studies were also found in fish such as channel catfish and Atlantic salmon [41]. These results suggest that *CART* has the function of regulating appetite. Further studies found that food intake decreased after intracerebroventricular injection of human *CART* fragments in goldfish [43], indicating that *CART* plays an appetite-suppressing role in these species. Taken together, *CART* and *POMC* are appetite suppressors. *Nucb2* also reduces food intake. The authors of [44] reported that intraventricular injection of nesfatin-1 could reduce food intake in mice. In 2012, Ref. [45] also found that intraventricular injection of 0.3 and 0.9 nmol of the nesfatin-1 fragment can significantly reduce the food intake of mice within 4 h after injection [46]. NPY and AgRP are secreted by NPY/AgRP neurons. To date, NPY has been studied and reported in most teleosts, including rainbow trout [47], zebrafish [48] and Siberian sturgeon [49]. A study showed that NPY regulates food intake [50]. The study found that the *NPY* mRNA expression level and protein release of fasting goldfish, *Schizothorax japonicus* and Siberian sturgeon increased significantly and had the opposite effect after refeeding [49,51]. In a rhythmic feeding test, NPY was highly expressed before feeding and significantly decreased after feeding [52]. After central injection of NPY, it was found that the animal’s foraging desire, food hoarding and food intake increased significantly, but this increase was transient and quickly returned to baseline over time. The cDNA of AgRP has been isolated and identified in a variety of fish, including *Schizothorax prenanti* [53], zebra fish [54], rainbow trout [55] and Siberian sturgeon [56]. Similar to *NPY*, *AgRP* mRNA expression and plasma AgRP concentration increased significantly under fasting conditions and decreased after refeeding [57]. In addition, intracerebroventricular injection of AgRP can effectively promote food intake [58]. The time effects of AgRP and NPY on regulating appetite are different. The time of NPY promoting appetite is shorter, while the time of AgRP is longer [59]. In addition, both POMC/CART and NPY/AgRP can act on melanocortin 4 receptor (MC4R) and regulate downstream pathways to influence appetite. A recent study in Siberian sturgeon has shown that mc4r expression can be inhibited by NPY injection [56]. The ablation of MC4R inhibited the activation of POMC neurons and promoted feeding of mice [60]. Thus, these unigenes, such as *POMC*, *CART*, *NPY*, *AgRP NUCB2*, *PYY* and *MC4R*, were selected as candidate genes in the present study. The qPCR results of the expression of these genes in successful and failed weaning sturgeon validated the accuracy of the RNA-seq.

To further understand these appetite regulators, the cDNAs of *POMC*, *CART*, *NPY* and *AgRP* were cloned. To the best of our knowledge, the sequences of *POMC*, *CART*, *NPY* and *AgRP* in Yangtze sturgeon have been obtained for the first time in this study. Subsequently, phylogenetic trees were constructed. There was no doubt that CART and POMC of Yangtze sturgeon were clustered with teleost fish, which was consistent with morphological classification. Phylogenetic trees of *NPY* and *AgRP* showed that *AgRP* of Yangtze sturgeons and Chinese sturgeons had higher homology with mammals or amphibians but lower homology with bony fishes such as zebrafish and Atlantic salmon. These results would be caused by the extra genome replication that occurred in other teleosts. Previous studies have shown that genome-wide replication occurred in teleost fish lineages 320–350 million years ago (MYA) [61,62,63]. Sturgeon, however, have been on Earth for 200–250 million years, so they are not part of this replication period. In addition, in some fish lineages, such as salmon and cyprinid fish, more genome replication occurs later. Cyprinid fish, in particular, have an extra copy of their genome, which may be responsible for differences in *NPY* and *CART* between sturgeon and other teleost fish.

To further investigate the effect of weaning on Yangtze sturgeon, qPCR in the brain of Yangtze sturgeon in different weaning phases was conducted. In the current study, *POMC* decreased in the early step of weaning but increased significantly on the 10th day of weaning. The expression of *CART* increased in the early stage but decreased in the middle and later stages. The expression pattern of *AgRP* was in contrast with that of *CART*, which was downregulated in the early stage but upregulated in the middle and later stages. The expression of *NPY* remained stable during weaning. These results were in contrast to a study conducted in rats, which indicated that *NPY* expression was evaluated, while *POMC*, *CART* and *AgRP* did not significantly change [17]. Our current results are also different from those of [64] from piglets. Nevertheless, the work of Peng, Dou, Liang, He, Liang and Shi [18] on Chinese perch has demonstrated that the substitution of live food will downregulate the expression of *POMC*, which is consistent with our present study. These results suggest that the appetite-regulating mechanism in mammals and teleosts is different. The present study also showed that the expression of both orexin factors and anorexin factors increased significantly except on the 1st day after refeeding with the microencapsulated diet. These appetite factors in the hypothalamus may serve as nutrient-sensing factors, so when live food appeared again, their expression was upregulated. Overall, the appetite of Yangtze sturgeon decreased in the early stage but increased in the later stage of weaning in our present study, and *CART* and *AgRP* played crucial roles in this period.

## 4. Material and Methods

### 4.1. Experimental Fish and Animal Welfare

All Yangtze sturgeon (*Acipenser dabryanus*) in the experiment were the second generation bred and provided by the Fisheries Institute, Sichuan Academy of Agricultural Sciences (SAAS), Sichuan Province. Yangtze sturgeon broodstock (first generation) was raised in filtered river water (20.5 ± 0.5 °C) with dissolved oxygen (DO) level > 5 mg/L under natural lighting. During the temporary breeding period, the sturgeons were fed with specific compound feed (longitudinal combined feed, Sichuan, China) and Tubificidae in a ratio of 1:1 with a feeding amount of 0.5% of body weight. For the induction of spawning, injections of luteinizing-hormone-releasing hormone analog (LRH-A2, Ningbo, China) were administered to the broodstock. Male sturgeon received a single injection at a dosage of 5 μg/kg. Female fish were subjected to a two-stage injection protocol, with the first injection at a dosage of 1 μg/kg, followed by a second injection at a dosage of 9 μg/kg after a 12-hour interval. Sperm and eggs were collected by gentle abdominal pressure. Fertilization was performed using the dry method under light-excluded conditions. To facilitate the detachment of the fertilized eggs, they were processed in a device equipped with talcum powder and an aeration system for de-adhesion. The fertilized eggs were then transferred to an incubator for hatching at the same temperature. Yangtze sturgeon after oral feeding (4 days post hatching, DPH) were fed with Tubificidae until 60 DPH. The experiments were performed by SAAS and Sichuan Agricultural University. All experimental procedures were approved by the Animal Care and Use Committee of SAAS and Sichuan Agricultural University (approval numbers: SAAS20190628 and DKY-S20190629).

### 4.2. Experimental Design

In the breeding process of sturgeon seeds, there is a weaning stage (the living bait is changed to artificial feed). Sturgeon (60 DPH) were fed with cut Tubificidae that was gradually replaced with a commercial microencapsulated diet at a rate of 10% per day for 10 days until complete replacement. Then, the sturgeon (70 DPH) were continuously fed with the microencapsulated diet twice a day until satiety. After weaning for a period of time (generally 160 days post hatching, DPH), some fish will have closed mouth behavior, which is characterized by a thin body, poor vitality and less than 70% intestinal filling degree after feeding for 30 min. To investigate whether these effects are related to appetite regulation in Yangtze sturgeon, 5 failed wean juvenile sturgeons and 5 successful wean juvenile sturgeon were selected separately for sampling in this study. After anesthetization, the filling degree was observed, and the body weight and body length of the fish were recorded. Then, the brain tissues were removed, washed with frozen normal saline, soaked in RNA storage preservation solution at 4 °C for 12 h and transferred to −80 °C. Subsequently, these brain tissues were used for RNA extraction and RNA-seq, and the brains of five sturgeon were mixed and sequenced.

Based on RNA-seq and reports on appetite regulation in teleosts, this study performed gene cloning. For cloning, five healthy juvenile sturgeon (224.56 ± 31.75 g) were hypothermally anesthetized, and the brain tissues were sampled and stored at −80 °C.

In addition, to further investigate whether weaning is influenced by appetite regulation, a weaning experiment was carried out in this study. All sturgeon were fed with cut Tubificidae to 60 DPH after oral feeding (4 DPH). Then, 360 sturgeon were randomly divided into 3 groups with three repetitions, in which 120 sturgeon (3 × 40) were still fed with cut Tubificidae to 70 DPH as the control group. A total of 120 sturgeon in the weaning group were fed compound meals that were fed a commercial microencapsulated diet (crude protein 50.0%, crude fat 8.0%, crude fiber 3.0% and ash 16%) from 61 to 70 DPH. The other 120 sturgeon of the refeeding group were fed a commercial microencapsulated diet to 65 DPH and refed with cut Tubificidae to 70 DPH. All fish were fed twice a day at 8:00 and 20:00. The water exchange volume is one-third of the water volume, and the water temperature is 20.5 ± 0.5 °C. After anesthetization with MS-222, brain tissues were sampled at 1, 3, 5, 6, 8 and 10 days after weaning in the control and weaning groups and similarly sampled at 6, 8 and 10 days after weaning in the refeeding group. During sampling, 2 fish were randomly selected at each time point for each repetition in each group (*n* = 6 = 3 × 2). The brain tissues were washed with frozen saline, soaked in RNA storage preservation solution at 4 °C for 12 h and transferred to −80 °C for RNA extraction and qPCR.

### 4.3. RNA-Seq

Total RNA was extracted with the Animal Tissue Total RNA Extraction and Purification Column Kit (Sangon Biotech, Shanghai, China). The extraction processes were conducted according to the manufacturer’s protocol. The concentration of total RNA was detected by a bioanalyzer (Agilent 2100, Agilent Technologies, Santa Clara, California USA) and 1% agarose gel electrophoresis, and only RNAs with RIN ≥ 7.0, OD 260/280 ≥ 1.8 and OD260/230 ≥ 1.5 were adopted for the next step. cDNA library construction and sequencing were conducted by Majorbio (Majorbio, Shanghai, China). To ensure the reliability of sequencing, SeqPrep (https://github.com/jstjohn/SeqPrep (accessed on 8 July 2011)) and Sickle (https://github.com/najoshi/sickle (accessed on 4 April 2012)) were used to filter the raw reads and remove the low-quality sequences (unknown nucleotides greater than 5%). After obtaining clean reads, de novo assembly was performed with Trinity (https://github.com/trinityrnaseq/trinityrnaseq/wiki (accessed on 25 May 2019)) to obtain the longest non-redundant unigene set, and then the unigenes were compared by six databases to obtain annotation information. The six databases are the following: NR (https://www.ncbi.nlm.nih.gov/refseq/about/nonredundantproteins/ (accessed on 24 August 2020)), Swiss-Prot (https://www.expasy.org/resources/swiss-model (accessed on 8 December 2019)), Pfam (http://pfam.xfam.org/ (accessed on 27 September 2018)), COG (https://www.ncbi.nlm.nih.gov/research/cog-project/ (accessed on 25 November 2020)), GO (http://www.geneontology.org (accessed on 29 May 2019)) and KEGG (http://www.genome.jp/kegg/ (accessed on 1 November 2018)).

Subsequently, RESM software (http://deweylab.github.io/RSEM/ (accessed on 27 June 2018)) was used to compare and estimate the expression abundance of the unigenes after assembly. After the read counts between different samples were standardized based on the TMP method, DEGseq software (1.60.0), was used to screen the differentially expressed genes (DEGs) according to *p* adjusted < 0.001 and |log2 (fold change)| ≥ 1. The unigenes were compared by RSEM using the transcripts per million reads (TPM) method. To further analyze the biological function of DEGs, GO and KEGG pathway functions were annotated and enriched for significantly upregulated and downregulated genes (*p* < 0.05).

### 4.4. Cloning and Sequence Analysis of POMC, CART, NPY and AgRP

Total RNA of the brains was extracted with the Animal Tissue Total RNA Extraction and Purification Column Kit (Sangon Biotech, Shanghai China). The quality of RNA was detected as mentioned above. The cDNA for cloning was prepared using the PrimeScript^TM^ RT Reagent Kit (037A, Takara, China). Primers for four critical appetite factors, *POMC*, *CART*, *NPY* and *AgRP*, were designed according to the relevant gene sequences of *Acipenser ruthenus* published in NCBI (GenBank accession numbers: XM_034019002, XM_034041740, XM_033993579 and XM_034041740, respectively) using Primer premier 5.0 and were synthesized by Sangon Biotech (Shanghai, China), as shown in Table 1. The PCR program was similar that in to our previous studies [65]. The PCR products were purified and ligated into the pMD-19T vector (Trans Gene Biotech, Beijing, China), and then they were introduced into competent *Escherichia coli* DH5α cells (Takara, Dalian, China) for sequencing (Sangon Biotech, Shanghai, China).

The cDNA fragment sequences of the cloned genes were compared using the website of NCBI (https://www.ncbi.nlm.nih.gov/ (accessed on 8 May 2020)). Sequence splicing was performed with DNAStar lasergene (https://www.dnastar.com/ (accessed on 8 May 2020)). Signal peptide cleavage sites were analyzed by SignaIP-5.0 (https://services.healthtech.dtu.dk/service.php?SignalP-5.0 (accessed on 9 May 2020)). Clustalx W (http://www.clustal.org/ (accessed on 9 May 2020)) was used for multiple sequence alignment analysis of amino acid sequences, MEGA 7.0 (https://www.megasoftware.net/ (accessed on 9 May 2020)) was used to construct the neighbor-joining trees and the analysis reliability was assessed by 1000 bootstrap replicates.

### 4.5. qPCR

RNA extraction and detection were performed as mentioned earlier. Then, cDNA was synthesized using the PrimerScript^TM^ RT Reagent Kit (Takara, Dalian, China), which was conducted according to the instructions. The expression of four candidate DEGs (*NUCB2*, *AgRP*, *POMC*, *CART*, *NPY*, *PYY*, *MC4R*, *JUNB*, *AKT*) was determined by qPCR to confirm the transcriptome-sequencing results. Moreover, four critical appetite factors, *POMC*, *CART*, *NPY* and *AgRP*, which were cloned in this study, were determined by qPCR to analyze their expression patterns in the weaning experiment. In a previous study, it was found that both elongation factor 1-alpha (*EF1-α*) and *β-actin* were stably expressed in the Yangtze sturgeon [66]. Thus, the target gene expression was normalized using these two housekeeping genes in this study. qPCR was also carried out as described in a previous study [65]. According to all standard curves, the primer amplification efficiencies of all investigated genes were 96.6–100.1% and 0.961 < R^2^ < 1.002, respectively. The target gene was normalized to the reference genes (geometric averaging of *β-actin* and *EF1-α* cycle threshold (Ct) value), and expression levels were compared using the relative Ct method (2^−ΔΔCt^) [67].

### 4.6. Statistical Analysis

The data in this study are presented as the mean ± SEM. SPSS 22.0 statistical software (SPSS Inc., Chicago, IL, USA) was used for statistical analysis. An independent between-variable *t*-test was performed to determine the significant differences between the two groups. *p* < 0.05 was considered to be statistically significant.

## 5. Conclusions

In conclusion, weaning failure will reduce the growth rate of Yangtze sturgeon. In addition, the expression of 126,855 genes was affected by weaning, including several appetite factors (*POMC*, *CART*, *AgRP*, *PYY* and MC4R) and the PI3K-AKT signal pathway, which are closely related to appetite regulation. Based on the results of RNA-seq, sequences of Yangtze sturgeon *POMC*, *CART*, *NPY* and *AgRP* were cloned. In the brain of Yangtze sturgeon, the mRNA expression of anorexin factor *CART* increased and that of orexin factor *AgRP* decreased in the early phase of weaning. In the middle and late phases of weaning, the expression of *CART* decreased and that of *AgRP* increased. In addition, *POMC* expression increased significantly only on the last day of weaning. The expression of these appetite factors indicates that the appetite of Yangtze sturgeon decreased in the early stage but increased in the later stage of weaning. The current study lays a foundation for further exploration of the appetite regulation mechanism in Yangtze sturgeon and other teleosts.

## Figures and Tables

**Table 1:** Sequences of primers for cloning and qPCR.

Primer Name	Primer Sequence	Applications
*POMC-F*	GGACCTCACCGCAGAATC	cDNA cloning
*POMC-R*	TAAACAAGGGCTTTGGCAG	
*CART-F*	ATTCCCGACTGTGGTTGAGA	
*CART-R*	ACAGTCACACAACTTGCCGAT	
*NPY-F*	ATTACCTCCTAAAGATGCGTT	
*NPY-R*	CACTACATCAATCTTATCACGC	
*AgRP-F*	GCTGGACAAGACCCAAGAT	
*AgRP-R*	CAGTAGCAGATGGCATTGAA	
*POMC-qF*	AGCACCACCCTTAGCGTTCT	qPCR
*POMC-qR*	ACCTCTTGTCATCCCGCCT	
*CART-qF*	CGACTGTGGTTGAGAGCCG	
*CART-qR*	GACAGTCACACAACTTGCCGAT	
*NPY-qF*	GCTGGCTACCGTGGCTTTC	
*NPY-qR*	GACTGGACCTCTTCCCATACCT	
*AgRP-qF*	AGGCTGTGCGTCTCAGTGTC	
*AgRP-qR*	GAATCGGAAGTCCTGTATCGG	
*NUCB2-qF*	TGGAGACAGACCAGCATTTCAG	
*NUCB2-qR * *PYY-qF* *PYY-qR* *MC4R-qF* *MC4R-qR* *JUNB-qF* *JUNB-qR* *AKT-qF* *AKT-qR*	GGCTCCGTAACCTGTTCACTTC AGGCAGAGGTATGGCAAGCG GGAGGGTCAGGAGACGGGAT ATGAAGAGAATCGCAGTCCT GGTGGAGAAAGAATGGTGC ACTCGTTTCTCTCTGCTTATGGC GCTCGTTCAAGTTCAGGCTCA CTGATGGCTCTTTCATAGGCTAC TGTTTGGCTTTGGTCGTTCT	
*β-actin-F*	CTGTTTCAGCCATCCTTCTTG	Reference genes
*β-actin-R*	TTGATTTTCATTGTGCTCGGT	
*EF1-α-F*	ATGTTCACAATGGCAGCGTC	
*EF1-α-R*	AAGATTGACCGTCGTTCCG	

## Data Availability

The Sequence Read Archive (SRA) has been deposited at GenBank in NCBI. The datasets generated during the current study are available in the NCBI GenBank repository, with accession number PRJNA955819. Please see link below for details (https://www.ncbi.nlm.nih.gov/bioproject/PRJNA955819) (accessed on 13 December 2024).

## Supplementary Materials

The following supporting information can be downloaded at: https://www.mdpi.com/article/10.3390/ijms26030950/s1.
