# Supplementary material for: Exploration of Appetite Regulation in Yangtze Sturgeon (Acipenser dabryanus) During Weaning
Source: Int J Mol Sci. 2025 Jan 23;26(3):950. doi: 10.3390/ijms26030950 (PMC11817240; doi:10.3390/ijms26030950)

Supplementary Table S1. Statistics of transcriptomic sequences from the two libraries.

Sample	Raw reads	Raw bases	Clean reads	Clean bases	Error rate(%)	Q20 (%)	Q30 (%)	GC content (%)
F_B	62910056	9499418456	62356550	9258226330	0.0247	98.18	94.35	46.25
S_B	53290740	8046901740	52743864	7832769909	0.0249	98.13	94.22	45

Supplementary Table S2. Statistical table of assembly result evaluation.

Type	Resource
Total transcripts number	126,855
Total unigenes number	82,151
Total sequence base	13705634
Largest	15,381
Smallest	201
Average length	1,080.42
N50	1,987
E90N50	2,573
GC percent	43.50
TransRate score	0.41746
BUSCO score	89.7% (3.0%)

Supplementary Figure S1. Statistical table of assembly result evaluation. Distribution of the annotated genes. (A) Unigene function annotated Veen diagram. (B) the species distribution of blast hits for each unigens.

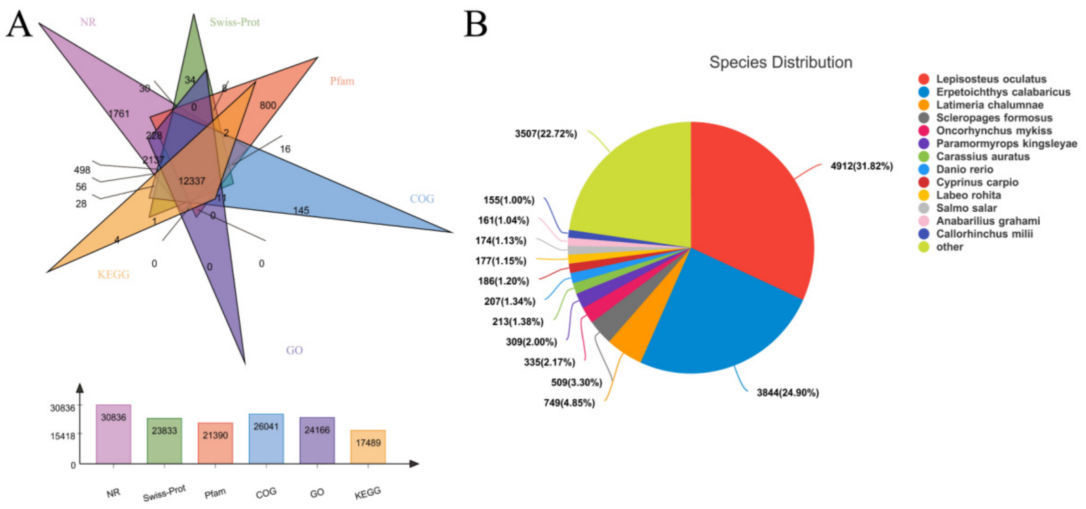

Supplement: Supplementary file 1 [file ijms-26-00950-s001.zip › ijms-3397678-supplementary.pdf]
